# Should We Shorten the Preclinical Curriculum? A Commentary on Accelerated Pathways in the Pass/Fail Era

**DOI:** 10.1177/23821205251395289

**Published:** 2025-11-14

**Authors:** James R. Burmeister, Ismail Zazay

**Affiliations:** 1 Department of Foundational Medical Studies, 159878 Oakland University William Beaumont School of Medicine, Rochester, MI, USA; 2 John Sealy School of Medicine, 12338 University of Texas Medical Branch, Galveston, TX, USA

**Keywords:** preclinical curriculum, medical education reform, USMLE step 1 pass/fail, clinical readiness, competency-based education, curriculum acceleration, foundational sciences, student perspective, educational equity, professional identity formation

## Abstract

As medical education evolves in response to the transition of the United States Medical Licensing Examination (USMLE) Step 1 exam to a pass/fail system, there is growing momentum to shorten the traditional two-year preclinical curriculum. This reflective piece, co-authored by two medical students, explores the motivations behind this shift, including the desire for earlier clinical exposure, reduced academic pressure, and a curriculum better aligned with long-term clinical competence. The authors acknowledge potential benefits, such as enhanced student engagement and improved relevance of foundational knowledge, but caution against a rushed transition. Risks include compromised scientific understanding, inequities among students with differing backgrounds, and insufficient time for professional identity formation. The article calls for thoughtful, evidence-based reform that ensures equity, supports foundational competence, and incorporates diverse student perspectives. Ultimately, while the shortening of preclinical years may offer advantages, it must be implemented carefully to avoid undermining the very foundations of medical education.

The ongoing trend toward shortening the preclinical curriculum in U.S. medical schools, especially after the United States Medical Licensing Examination (USMLE) Step 1 examination shifted to pass/fail scoring, raises important questions about foundational science, student equity, and long-term clinical readiness. In this commentary, we synthesize recent research and national trends to evaluate whether preclinical education should be shortened and under what conditions, arguing that reforms must prioritize evidence, integration, and equity.

As third- and fourth-year medical students entering and progressing through our clerkship phases, we have witnessed firsthand how rapidly medical education is evolving. One of the most significant changes is the movement to shorten the traditional two-year preclinical phase, often to as little as 12 to 15 months. Although the 2022 Step 1 pass/fail change catalyzed reform, discussions about integrated and shorter preclinical models were already emerging before this transition in national policy analyses and academic research.^1–3^

The once-feared three-digit Step 1 score is now obsolete, and along with it much of the pressure students felt they must face to “learn to the test.” However, early reports indicate that Step 1 pass rates have declined since the scoring change, suggesting new challenges in knowledge retention and preparedness for clinical responsibilities.^4^

The move to a pass/fail format was intended to reduce high-stakes stress, unhealthy competition, and inequities in residency selection by promoting a more holistic evaluation of students focused on competency rather than numerical scores.^5^ National medical education organizations advocated for this change to foster learning and professional growth over test performance. Interestingly, some students have now reversed the traditional exam sequence, taking Step 2 before Step 1 because Step 2 remains numerically scored and has become the primary standardized metric for many residency programs.^6^

Several prominent medical schools, including New York University (NYU), Duke University, and the University of Pennsylvania (Penn), have already shortened their preclinical phases, but the long-term outcomes of these reforms remain uncertain.^3,7^ Evidence to date suggests that while curricular acceleration can enhance early clinical exposure and engagement, it must be balanced with robust reinforcement of foundational science, equitable access, and sustained professional identity formation. These considerations form the foundation of the discussion that follows.

Notably, discussions have been presented about this same topic even prior to the USMLE Step 1 going pass/fail. Scoring reform calls to reduce the amount of time required for medical training have been highlighted in the literature as early as 2012, including discussions in support of more immediately reducing the total time to obtain the MD degree. Emanuel and Fuchs asserted that medical training in the U.S. is overly lengthy relative to other countries and agreed that one way to shorten training is to remove some of the redundant preclinical content, while directly integrating basic science with clinical experiences, and enhance the residency pathway.^8^ Other articles similarly expressed the view that the time-based model often fails to meet society's needs and posed that a competency-based progression, instead of some fixed number of years, would allow the student to master the content in succession, while achieving required competencies.^9^ Together, this work provides a context for moving forward: does one wish to reduce time only for efficiency, or the ability to recast training to produce core competencies in a different and more integrated and relevant manner?

Recent reviews emphasize that the effect of shortening preclinical time depends on robust integration of foundational sciences throughout the entire medical curriculum, and caution that evidence on long-term outcomes remains limited.^3,10^

But as two students, one beginning and one progressing through clerkships, who’ve been through the preclinical gauntlet and are applying that knowledge on the wards, we’ve found ourselves asking: Should we really shorten the preclinical years? What might we gain, and what might we lose?

## Why the Shift Sounds Appealing: Reconnecting With the “Why” Behind the “What”

There's no doubt that the appeal of an accelerated preclinical curriculum is real, especially to students eager to move beyond textbooks and into authentic clinical environments.

The traditional model often emphasizes the “what”, memorizing mechanisms, diseases, and biochemical pathways, but can obscure the “why”: why this knowledge matters, how it connects to patient care, and what kind of doctor we want to become. When you're buried in lecture slides and spaced repetition apps, it's easy to forget that all this information is supposed to help you treat human beings.

By shortening the preclinical phase, schools aim to reconnect students with that deeper purpose. Earlier clinical exposure provides context and meaning to the information we’re learning. Seeing patients with the very diseases we just studied transforms abstract knowledge into something tangible and motivating. You begin to think less like a student and more like a clinician.

Additionally, removing the pressure of scoring high on Step 1 allows educators to restructure the curriculum around competency and integration, rather than solely test performance. The hope is that this will reduce student burnout, promote more meaningful learning, and free up time for research, electives, and Step 2 preparation, all of which now play an outsized role in residency selection.^3,5^

This observation has been supported by previous work as well. For example, Dickinson et al. (2020) showed that medical students are more engaged with biomedical sciences when it is applied to clinical practice.^11^ In their study, students discussed how linking interpersonal experiences with physiology, immunology, biochemistry, and all their other content, to real patient cases, turned narrow knowledge into a large scale of clinical knowledge, with students being able to engage for longer periods and with better focus. Students’ engagement with content was also strengthened, leading to more mindful learning in their studies, such as being able to link specific illness types/conditions with their own individual patient populations. Rather than knowing only the molecular pathways in isolation, students shift their perspective to engage with those pathways in the context of their patient. This integration created physiological identification, rather than biomedical sciences being an obstacle and something to “get past” to become a doctor; mastering biomedical science becomes an aspect of what it means to be a physician and part of their identity. In other words, by embedding the clinical application within the entire preclinical phase, students gain cognitive benefits in retention and reasoning but also purpose and belonging.

Recent reports from the Association of American Medical Colleges and Academic Medicine indicate that curricular integration and early clinical exposure are strongly associated with greater student engagement, higher satisfaction, and more positive perceptions of relevance, as long as foundational sciences remain meaningfully linked to clinical experience.^3,12^

## But Here's the Risk: Foundations Still Matter

Despite the potential benefits, shortening the preclinical phase comes with real risks, risks that are especially evident once you step onto the wards.

First and foremost: a strong clinical student still needs a strong scientific foundation. Even if Step 1 is pass/fail, the material it covers remains vital to understanding patient physiology, pathophysiology, pharmacology, and beyond. Clinical reasoning depends on that foundation, not just for exams, but for real-world decision-making.^13^

When created initially, the Step examinations were not intended to rank students for residency but were instead used to ensure standardized competency among medical licensure.

Step 1 assessed basic science knowledge needed to progress into clinical training whereas Step 2 evaluated the application of that knowledge in supervised practice; and Step 3 tested readiness for independent practice. The later use of these exams as competitive filters for residency selection was a deviation from their original purpose. Recognizing this history reminds us that Step 1 being pass/fail does not diminish its importance as a competency milestone.^6^

Data suggest that students who perform well on Step 1 tend to perform well on Step 2, which many programs now weigh heavily in residency applications.^5^ A 2021 cohort study found that students at one U.S. medical school who experienced a shortened preclinical curriculum performed significantly lower on the National Board of Medical Examiners (NBME) surgery shelf examination compared to those with traditional two-year preclinical training, regardless of clerkship timing, raising concern about knowledge transfer and preparedness.^14^ If students breeze through the preclinical years or treat them as “less serious” due to the Step 1 scoring change, they may find themselves at a disadvantage when it's time to take the next big test, and, more importantly, when it's time to care for patients.

Additionally, the preclinical years are not just academic. They offer time to develop study skills, build professional identity, reflect on ethics, and adapt to the mental and emotional demands of medicine.^15^ Compressing this period into a sprint could leave students, especially those from non-traditional or underrepresented backgrounds, without the time or support they need to succeed.

Another issue to identify is equity. If preclinical time is shortened, admissions policies and prerequisite expectations must evolve accordingly; otherwise, students from nontraditional or liberal-arts backgrounds may be disadvantaged. Without adjustments to admissions requirements and premedical coursework, accelerated curricula risk worsening existing inequities.^16^

Currently, there is limited high-quality, long-term data on whether accelerated curricula impact clinical competence, specialty choice, burnout, or patient care outcomes after graduation. Many published studies are single-site or focus on short-term metrics such as course evaluations and exam scores. Ongoing multi-institutional and longitudinal studies are needed to address these gaps in evidence.^3,10,17^

## What the Data Show, and What's Still Missing

Some highly ranked schools have already shortened their preclinical curricula, such as NYU, Duke, and Penn. The use of additional time created by shortening the preclinical phase varies between institutions. In some programs, this time is dedicated to more clinical rotations or electives, while in others, it may be allocated to research, Step 2 preparation, or flexible learning. As a result, it is not always clear that total clinical training time increases; an area that warrants further study.

However, the broader evidence is still limited. Most available studies focus on short-term outcomes, shelf exam scores, course evaluations, and Match results, but we still don’t fully understand the long-term effects on clinical preparedness, specialty choice, burnout, or residency performance. These are complex outcomes that require more than just surveys and grades to assess.^10,17^

## Global Trends

Globally, six-year medical school models dominate, with students beginning training directly after secondary education. Countries across Europe and Asia have long emphasized earlier clinical exposure integrated with biomedical sciences. U.S. programs such as University of Missouri-Kansas City (UMKC) and Howard offer six- or seven-year BS/MD pathways that echo this model. Although these programs differ from standard U.S. models, comparative studies suggest that the key to success lies in longitudinal integration of basic sciences rather than time alone.^18^

## Our Perspective: Don’t Rush the Roots

While personal experiences offer valuable context, recommendations must be grounded in both literature and outcome data.

As two students standing on the edge between the classroom and the clinic, we’ve come to appreciate just how much the preclinical years prepared us, and how much more we might still need to revisit. It's tempting to think that those years were just a means to a test. But in reality, they were the roots of everything we’re doing now: taking histories, understanding labs, interpreting findings, and formulating differentials.

It's true that not every detail sticks, and yes, the system can be improved. But compressing the preclinical curriculum too aggressively may rob students of the time they need to actually understand medicine, not just pass an exam.

## Final Thoughts: A Promising Idea, But One That Demands Caution

Based on our review of the recent literature, national policy statements, and our experience as learners, we propose that shortening the preclinical curriculum may be beneficial only if reforms include robust integration of foundational science throughout all phases of training. Schools must proactively address equity by supporting students from diverse educational backgrounds and evaluating the impact of reforms on access and success. Further research is needed to determine the long-term effects of these curricular changes on clinical competence, specialty choice, well-being, and patient outcomes. Student perspectives, along with rigorous outcome measurement, should remain central to any reform process.

The move toward earlier clinical exposure is a good thing. But that doesn’t mean we should devalue the foundational science or treat the preclinical years as obsolete now that Step 1 is pass/fail. The main challenge is not simply “how short” the preclinical years should be, but how effectively the curriculum integrates foundational sciences with clinical reasoning across all four years.

Further multi-institutional research and inclusion of student voices are essential to ensure reforms remain evidence-based and equitable. And if those things happen, then yes, shortening the preclinical curriculum may very well be a worthwhile evolution in medical education.

In summary, accelerated preclinical curricula must be implemented with caution, grounded in evidence, and focused on integration, equity, and long-term student outcomes.
